# Prevention of noncommunicable diseases by interventions in the preconception period: A FIGO position paper for action by healthcare practitioners

**DOI:** 10.1002/ijgo.13331

**Published:** 2020-09-07

**Authors:** Chandni Maria Jacob, Sarah Louise Killeen, Fionnuala M. McAuliffe, Judith Stephenson, Moshe Hod, Ivonne Diaz Yamal, Jaideep Malhotra, Edgar Mocanu, H. David McIntyre, Anne B. Kihara, Ronald C. Ma, Hema Divakar, Anil Kapur, Rui Ferriani, Ernest Ng, Laurie Henry, Zephne Van Der Spuy, Zev Rosenwaks, Mark A. Hanson

**Affiliations:** ^1^ Institute of Developmental Sciences Faculty of Medicine University of Southampton Southampton UK; ^2^ NIHR Southampton Biomedical Research Centre University Hospital Southampton Southampton UK; ^3^ UCD Perinatal Research Centre School of Medicine University College Dublin National Maternity Hospital Dublin Ireland; ^4^ Elizabeth Garrett Anderson Institute for Women’s Health University College London London UK; ^5^ Mor Comprehensive Women’s Health Care Center Tel Aviv Israel; ^6^ FIGO Pregnancy and Non‐Communicable Diseases Committee International Federation of Gynecology and Obstetrics London UK; ^7^ Faculty of Medicine University Militar Nueva Granada Bogota Colombia; ^8^ Fertility Center Procreation Medicamente Asistida Bogota Colombia; ^9^ FIGO Committee for Reproductive Medicine, Endocrinology, and Infertility International Federation of Gynecology and Obstetrics London UK; ^10^ Malhotra Nursing and Maternity Home Agra India; ^11^ Rainbow Hospital Agra India; ^12^ RCSI Department of Reproductive Medicine Rotunda Hospital Dublin Ireland; ^13^ Mater Research The University of Queensland South Brisbane Qld Australia; ^14^ African Federation of Obstetricians and Gynaecologists Khartoum Sudan; ^15^ Department of Obstetrics and Gynecology School of Medicine University of Nairobi Nairobi Kenya; ^16^ Department of Medicine and Therapeutics The Chinese University of Hong Kong Hong Kong SAR China; ^17^ Hong Kong Institute of Diabetes and Obesity The Chinese University of Hong Kong Hong Kong SAR China; ^18^ Divakar’s Specialty Hospital Bengaluru India; ^19^ World Diabetes Foundation Bagsværd Denmark; ^20^ Ribeirão Preto Medical School Human Reproduction Sector Department of Gynecology and Obstetrics University of São Paulo São Paulo Brazil; ^21^ Department of Obstetrics and Gynecology Li Ka Shing Faculty of Medicine University of Hong Kong Hong Kong SAR China; ^22^ Department of Gynecology and Obstetrics Centre de Procréation Médicalement Assistée (CPMA) University of Liège CHR de la Citadelle Liège Belgium; ^23^ Department of Obstetrics and Gynecology University of Cape Town Groote Schuur Hospital Cape Town South Africa; ^24^ The Ronald O. Perelman and Claudia Cohen Center for Reproductive Medicine Weill Cornell Medicine New York NY USA

**Keywords:** FIGO, Noncommunicable diseases, Nutrition, Obesity, Perinatal health, Preconception, Pregnancy

## Abstract

With the increase in obesity prevalence among women of reproductive age globally, the risks of type 2 diabetes, gestational diabetes, pre‐eclampsia, and other conditions are rising, with detrimental effects on maternal and newborn health. The period before pregnancy is increasingly recognized as crucial for addressing weight management and reducing malnutrition (both under‐ and overnutrition) in both parents to reduce the risk of noncommunicable diseases (NCDs) in the mother as well as the passage of risk to her offspring. Healthcare practitioners, including obstetricians, gynecologists, midwives, and general practitioners, have an important role to play in supporting women in planning a pregnancy and achieving healthy nutrition and weight before pregnancy. In this position paper, the FIGO Pregnancy Obesity and Nutrition Initiative provides an overview of the evidence for preconception clinical guidelines to reduce the risk of NCDs in mothers and their offspring. It encourages healthcare practitioners to initiate a dialogue on women’s health, nutrition, and weight management before conception. While acknowledging the fundamental importance of the wider social and environmental determinants of health, this paper focuses on a simple set of recommendations for clinical practice that can be used even in short consultations. The recommendations can be contextualized based on local cultural and dietary practices as part of a system‐wide public health approach to influence the wider determinants as well as individual factors influencing preconception health.

## Abstract 2

This FIGO position paper calls for clinicians to act on the need to promote healthy nutrition and BMI in the preconception period to prevent noncommunicable diseases.

## 1 Background

Definitions of the preconception period vary from “3 months before conception”^1^ to “a minimum of 1–2 years before the initiation of any unprotected sexual intercourse that could possibly result in a pregnancy”.^2^ More recently, the *Lancet* series on preconception health called for a new definition that considers multiple perspectives: biological (days to weeks before embryo development); individual (weeks to months before pregnancy); and public health (months to years).^1^

Preconception care is defined as “a set of interventions that aim to identify and modify biomedical, behavioral, and social risks to a woman's health or pregnancy outcome through prevention and management, emphasizing those factors that must be acted on before conception or early in pregnancy to have maximal impact”.^3^ This includes care provided regardless of pregnancy status or intention.^4^ A limitation in delivering preconception care has been the focus on women and couples motivated to conceive. For example, early marriage and early age at childbirth are common in low‐ and middle‐ income countries (LMICs) such as India.^5^ In addition, even in high‐income countries, a significant proportion of pregnancies are unplanned: 45% of pregnancies in the UK.^6^ Healthcare professionals often meet women with different perspectives on pregnancy^7^: “potential” (sexually active individuals without effective contraception or contraceptive failure); “intentional” (men and women making a conscious decision to conceive); and “public health” (a wider range of individuals and couples not sexually active). With the growing realization that efforts to optimize health of women and children should begin before conception, healthcare practitioners must be aware of these perspectives.

## 2 Preconception Health: An Opportunity to Prevent Noncommunicable Diseases (NCDs)

Globally, there has been a rapid rise in obesity among women of reproductive age, with almost 21% of women in the world predicted to be obese by 2025.^8^ In high‐income countries such as the USA, the prevalence of obesity in women aged 20–39 years was 31.8% in 2011–2012.^9^ With the nutritional transition in LMICs, overweight and obesity prevalence is increasing, for example in South Africa, China, and Brazil.^10^, ^11^ Thus, more women now enter pregnancy with excess weight and are subsequently at a higher risk of complications such as pre‐eclampsia, gestational diabetes (GDM), and macrosomia, and their children have an increased risk of obesity.^12^ Similarly, undernutrition before pregnancy (being underweight or micronutrient deficient) can lead to adverse outcomes such as low birth weight and intrauterine growth restriction, neural tube defects, and preterm delivery.^13^ Impaired maternal and paternal nutrition in the preconception period can affect embryonic development with long‐term consequences for the next generation.^14^ Box 1 summarizes key risk factors in the preconception period and maternal and fetal outcomes.^12^, ^14^, ^15^

Box 1 **Intermediary risk factors in the time around conception and effects on future noncommunicable diseases (NCDs) in mother and offspring.**

**Table:** 

Periconceptional risk factors for future NCDs	Perinatal complications	Risk to offspring	Long‐term impact on mother and offspring
Overweight and obesity Pre‐existing diabetes mellitus Smoking Polycystic ovarian syndrome High blood pressure Paternal factors such as suboptimal nutrition Micronutrient deficiencies (e.g. iron, folate, vitamin D) Underweight/undernutrition	Before pregnancy: reduced fertility, pregnancy loss Gestational diabetes Pre‐eclampsia, gestational hypertension Preterm birth	Altered birthweight (e.g. low birthweight, small for gestational age, macrosomia) Congenital anomalies (e.g. neural tube and cardiac defects) Increased adiposity in infancy and childhood	Cardiometabolic disorders Neuro‐developmental issues Childhood obesity and increased risk of type 2 diabetes/prediabetes Increased risk of type 2 diabetes in mother

Adverse conditions in critical periods before, during, and after pregnancy can have lasting effects on the child’s physical and neurological growth and development. This, in turn, can affect future outcomes such as education and economic productivity.^16^ Higher preconception body mass index (BMI, calculated as weight in kilograms divided by the square of height in meters) carries a significant risk for excess gestational weight gain in early pregnancy^17^ and childhood obesity,^18^ perpetuating the intergenerational cycle of NCDs. Growing evidence shows that dietary and lifestyle interventions to reduce excess gestational weight gain and improve birth outcomes may be inadequate.^19^ As the challenge to manage maternal obesity intensifies, greater emphasis on prevention is needed and support should be available for weight loss before pregnancy. This can have added benefits for women with subfertility who are trying to conceive, and those with comorbidities such as polycystic ovarian syndrome (PCOS) or pre‐existing diabetes.

Preconception interventions and counselling during clinical visits have shown very clear benefit in the prevention of neural tube defects and reducing the risk of fetal alcohol syndrome.^20^, ^21^ Increasing evidence suggests that preconception care can help modify dietary and physical activity behaviors and optimize medical conditions, such as diabetes, and reduce sequelae such as congenital anomalies.^22^, ^23^ Despite this, dedicated preconception clinics are rare in most countries. In addition, as a significant proportion of pregnancies are unplanned, experts and healthcare organizations have called for maximizing routine contact between healthcare practitioners and young women.^22^ This means that in many cases, the onus for preconception care is on healthcare practitioners who see women in their routine practice, for reasons other than pregnancy planning. Elements of preconception care can occur whenever a healthcare practitioner meets a woman of childbearing age and this would include offering support for pregnancy planning or contraception counselling, and addressing nutrition and weight management.^22^, ^24^, ^25^

## 3 Aim

This FIGO position paper summarizes key evidence and international guidelines from predominant areas of preconception health related to NCDs, nutrition, and obesity. The target audience of the paper includes all healthcare practitioners, healthcare delivery organizations, and public health policymakers. The authors provide a clear set of simple recommendations to increase awareness about the importance of preconception counselling among healthcare practitioners who meet women in the reproductive age group (including obstetricians and gynecologists, general practitioners, midwives, community health workers, pediatricians, nutritionists, etc), irrespective of the reason for the consultation. While this paper focuses on providing recommendations for good clinical practice, FIGO acknowledges that obesity and malnutrition are chronic conditions influenced by lifestyle, health behaviors, public health policies, and other factors outside the clinical domain (i.e. the social and environmental determinants of health).^26^ This is not a systematic review of all clinical guidelines for preconception care globally. Supporting information S1 provides a list of the available guidelines based on key international documents from maternal health organizations to aid the development of country‐specific strategies. We recommend that healthcare practitioners refer to the guidelines presented in Box 2 and contextualize them to their settings for routine practice.

Box 2 **Recommendations for clinicians by the FIGO Pregnancy and Non‐Communicable Diseases Committee and the FIGO Committee for Reproductive Medicine, Endocrinology, and Infertility to support the prevention of NCD risk factors in the preconception period.**

1. Preconception consultations should include the measurement of height and weight and the calculation of body mass index (BMI, calculated as weight in kilograms divided by the square of height in meters). Where appropriate, all women should be encouraged to attain a BMI as close to the normal range (18.5–24.9) as possible before conceiving.
2. All women who have a BMI greater than 30 should be counselled about the risks of obesity for their health and that of their baby.
3. Women who are underweight before pregnancy (BMI less than 18.5) should be counselled about the risk of being underweight during pregnancy along with the benefits of good nutrition as relevant to their socioeconomic status. Where relevant, women who are underweight should be screened for suspected eating disorders and treated as needed.
4. Counselling for physical activity should be provided when required. Prepregnancy, during pregnancy, and postpartum, where possible, women should exercise moderately for at least 30 minutes a day, 5 days a week, or achieve a minimum of 150 minutes of moderate exercise per week.
5. Clinicians should support women with known pre‐existing diabetes to achieve glycemic control (HbA1c <6.5%) before pregnancy along with optimal weight management and dietary advice. When feasible and indicated (e.g. type 1 diabetes mellitus), screening for thyroid dysfunction and coeliac disease should be performed.
6. Chronic conditions such as high blood pressure and polycystic ovarian syndrome should be optimally managed with medication appropriate for pregnancy as required before conception. Women should be counselled regarding the risk of cardiometabolic comorbidities during pregnancy.
7. Folic acid: to ensure protection against neural tube defects, all women of reproductive age are advised to consume 0.4 mg (400 µg) of synthetic folic acid daily, obtained from fortified foods and/or supplements. For all women planning a pregnancy, a dietary supplement of at least 0.4 mg (400 µg) of folic acid per day is recommended at least 1 month before conception and continuing during the first trimester of pregnancy.
8. Women at a higher risk of neural tube defects (e.g. on anticonvulsant medication, with prepregnancy diabetes mellitus, a previous child or family history of neural tube defects, BMI of 30 or greater) wishing to become pregnant should be advised to take at least 4 mg folic acid as a dietary supplement daily, starting at least 1–3 months before conception and continuing during the first trimester of pregnancy.
9. Nutritional deficiencies (e.g. iron, iodine, and vitamin D) should be assessed and treated and advice given as appropriate.
10. Where applicable, discussion on nutritional risks should include the diet and health of the partner too.

## 4 Barriers and Opportunities for Engaging with Women in the Preconception Period

Despite mounting evidence on the importance of the preconception period, the translation of guidelines into clinical and public health practice remains inadequate.^21^, ^27^ Opportunities for preconception care are also underutilized by healthcare practitioners, often due to time constraints during appointments, the lack of resources to discuss preconception care, and competing priorities during a consultation.^28^ Evaluation of the implementation of guidelines for obesity prevention in the general population has shown that recommendations for nutrition and weight loss are not regularly adopted by healthcare practitioners.^29^ This is influenced by perceptions of the causes of obesity, especially that it results from personal behavioral choice. A negative outlook regarding the effectiveness of behavioral interventions along with the healthcare practitioner’s understanding of the scope of their professional responsibility can also influence the delivery of preconception care.^29^, ^30^ Women have reported feeling stigmatized and offended after consultations, especially when the risks of obesity were discussed without offering structured support to address the issue,^31^ and this can lead to healthcare avoidance.^32^ Lack of planned nutrition discussions during the consultation, low priority for lifestyle and weight management, and a perceived lack of training and skills often hinder healthcare practitioners from discussing weight management, diet, and physical activity with patients.^33^ However, healthcare practitioners in maternity services are a group perfectly positioned to discuss preconception nutrition. Though a large proportion of women seek information related to nutrition on the internet, they consider clinicians as reliable sources of information and hence having conversations related to diet and lifestyle in the clinic can help overcome other barriers such as misinformation and poor‐quality nutritional information on the internet.^34^

Overall, community awareness of the benefit of preconception health and nutrition can be low,^30^ with most women, especially in LMICs, seeking medical care once they are already pregnant.^1^, ^13^ “Generally healthy” women in the population often do not engage with sexual and reproductive health care or primary care for preconception advice or to discuss pregnancy intentions, thus missing out on important information, increasing unplanned pregnancy and the risk of adverse maternal and fetal outcomes.^35^ Conversely, it has been reported that women who planned pregnancy were more likely to take folic acid supplementation, access information, and get appropriate immunizations compared to those not planning pregnancy and who had limited healthcare engagement.^36^

Poor understanding of health issues and a reduced capacity to use health information effectively are additional barriers to effective translation of health messages and adoption of healthy behaviors. To help overcome this issue, studies have suggested that healthcare practitioners utilize clear communication and plain language techniques with all women and their partners who approach them for nutrition counselling.^37^ However, to support behavior change, passive provision of information in the hospital setting may not be enough, as seen from multiple weight loss trials.^22^ Active strategies such as discussion and counselling are suggested, along with techniques such as motivational interviewing and healthy conversation skills, which show promise among healthcare practitioners for inducing lifestyle behavior change.^38^, ^39^

While several public health models of preconception care delivery have been described, most agree that a patient‐centered approach is needed as part of a continuum of care, taking into consideration the woman’s socioeconomic circumstances and encouraging the involvement of her partner when suitable.^1^, ^20^ Though dedicated preconception care clinics have great value in improving maternal and fetal outcomes, people attending such services often consist of highly motivated individuals who are planning a pregnancy. Such services also require investment in staff education, time, and resources.^40^ Discussions in antenatal visits are often limited to fetal health and immediate pregnancy outcomes. Including preconception care in routine visits thus provides an opportunity to improve women’s overall health and nutrition across the life course using a woman‐centered approach.^41^ For example, the Before, Between, and Beyond Pregnancy program is the largest preconception health programme in the USA, which aims to make preconception care a standard practice using every encounter with women to address risk factors prior to pregnancy that could influence birth outcomes.^42^ It provides information for patients and includes resources for clinicians on preconception counselling including nutrition, alcohol consumption, screening for obesity, and assessing patient readiness for weight loss.

In summary, FIGO recommends that healthcare practitioners make the most of every contact with women in this period to initiate the conversation about nutrition and weight management using opportunities such as postnatal care, endocrinology and diabetes clinics, and contraception services. Previous reviews have shown that women who received preconception or interconception care had improved outcomes such as increased intake of folic acid and other supplements, lesser gestational weight gain, increased weight loss in the postpartum period, and GDM risk reduction along with reduced risk of small‐for‐gestational age babies.^22^, ^23^ Summarized below are key NCD, weight, and nutrition‐related issues in the perinatal period that could be addressed during preconception visits and clinical guidelines for the same.

## 5 Achieving a Healthy Weight Before Pregnancy and Preventing Excess Gestational Weight Gain

It is estimated that over 50% of women who become pregnant are overweight or obese.^43^ While antenatal interventions and counselling for appropriate gestational weight gain are crucial, efforts must be made to support women in achieving a healthy weight before and after pregnancy. However, it must be noted that weight loss during pregnancy is not currently recommended. Several national guidelines have recommended that consultations with women in the preconception period should include a discussion on achieving a normal BMI (18.5–24.9), thus giving them an early opportunity to discuss potential risks and management of weight with a healthcare practitioner.^44^, ^45^, ^46^ Furthermore, healthcare practitioners are advised to convey the obstetric risks of being under‐ or overweight during pregnancy. Though improved outcomes are seen after a weight loss of about 5%–10% of original body weight in women with overweight or obesity, goals for weight loss need to be personalized considering the woman’s circumstances and any weight loss before pregnancy may be beneficial.^47^ Healthcare practitioners also need to consider the guidelines in their regions while discussing the risks of NCDs. For example, guidelines in some Asian countries have used lower BMI cut‐off points than in other populations.^45^ Strategies for improving BMI before pregnancy include behavioral strategies combined with dietary modification and physical activity. An important point for consideration is that improvement in nutrition and physical activity will improve women’s overall health and well‐being and future pregnancy, even if clinically significant weight loss is not achieved. Overall, evidence is stronger for dietary interventions and diet supplemented with exercise compared to physical activity alone, though the latter has additional cardiovascular benefits.^48^

Bariatric surgery is a recommended treatment for women with marked obesity; however, the BMI cut‐offs vary depending on the existence of comorbidities and between countries.^45^, ^49^ Although recommendations for the safe minimal period after metabolic surgery differ, with most guidelines recommending pregnancy at least 12–18 months after the surgery, conception after surgery is best delayed until weight has stabilized.^50^ Being underweight also has adverse obstetric and neonatal outcomes, such as low birth weight, placing the offspring at a higher risk of NCDs in the future.^16^ Screening for low BMI must be done where the local prevalence of undernutrition due to infections, food insecurity, and poverty is high. However, women who are underweight should also be screened for eating disorders if suspected and treated as necessary.^45^, ^51^

It is crucial that risks and strategies for weight loss or gain are communicated in a supportive and nonstigmatizing manner, setting realistic goals for weight management with appropriate referrals to a dietician/nutritionist when indicated.

Healthcare practitioners should use resources such as the FIGO Nutrition Checklist (supporting information S2) as a tool to address weight in practice,^52^, ^53^ the “Think Nutrition First” guidelines,^13^ and the “Management of Prepregnancy, Pregnancy, and Postpartum Obesity from the FIGO Pregnancy and Non‐Communicable Diseases Committee: A FIGO (International Federation of Gynecology and Obstetrics) Guideline” for further information.^47^

## 6 Nutrition and Micronutrient Supplementation

A healthy diet before conception may reduce pregnancy complications such as GDM and hypertension.^54^, ^55^ The nutritional guidelines for women contemplating pregnancy show great diversity between countries and we recommend that local dietary practices based on personal or religious beliefs are taken into consideration when providing nutrition advice. While dietary advice to consume more fruit and vegetables and wholegrains is common across most countries, certain countries have guidelines for particular food items, such as restricting coffee (Italy) and avoiding specific types of fish (Sweden, Italy).^56^

Most countries have guidelines for folic acid supplementation for women planning a pregnancy, and some countries include women not using contraception. Folic acid (at least 0.4 mg daily) should be taken for a minimum of 1 month before conception and the first 3 months of pregnancy. Where there is an increased risk of neural tube defects (anticonvulsant medication, pre‐existing diabetes mellitus, previous child or family history of neural tube defects, BMI >30), consideration should be given to using a higher dose (at least 4 mg daily).^57^ However, the guidelines for folic acid may vary in some countries, for example in the UK 5 mg folic acid is recommended for women with a BMI above 30 who are planning pregnancy.^51^

Micronutrient deficiencies (folate, iron, and vitamin B12) can result in issues such as anemia, with severe consequences in pregnancy such as spontaneous abortion, low birth weight, and contributing to perinatal and maternal mortality globally.^13^ Routine supplementation of nutrients varies between countries, likely due to differences in diet based on access, availability, and cultural or societal factors. The specific nutritional deficiencies to be evaluated in routine care must therefore be considered in the context of the nutritional status of the relevant population and reviewed based on the healthcare practitioner’s practice. In India, for example, approximately one‐third of women are vegetarian or have low consumption of meat, poultry, eggs, and fish, placing them at risk of iron and vitamin B12 deficiency.^13^ It is therefore recommended that all women in the preconception period be screened for anemia. In addition, Indian guidelines recommend weekly supplementation with iron (100 mg) and folic acid (500 µg) with deworming medication (400 mg albendazole) for all women in the preconception period.^45^ Dietary iodine supplementation (150 µg) before a planned pregnancy is recommended in Australia and New Zealand.^49^ In countries where there is low habitual intake of vitamin D or where the potential for endogenous production is limited due to location or skin covering, women of reproductive age may have vitamin D deficiency.^13^ This is especially important for women with obesity who are at high risk of vitamin D deficiency due to sequestration of the vitamin in adipose tissue.^46^ Though the evidence as to whether routine vitamin D supplementation improves maternal and offspring outcomes remains inconclusive, we recommend that, when deficiency is suspected, healthcare practitioners advise supplementation as appropriate. Women who have undergone bariatric surgery will require additional supplementation (e.g. vitamin B12, other vitamins, trace elements) and hence screening and treatment must be provided appropriately.^58^ Detailed guidance on micronutrient supplementation in the perinatal period has been published by FIGO’s Working Group on Good Clinical Practice in Maternal–Fetal Medicine.^57^

## 7 Management of Diabetes and Preconception Prevention of GDM

FIGO recommends that preconception care should include risk assessment for GDM for all women along with assessment for risk factors such as obesity.^59^ This aims to establish pre‐existing or undiagnosed diabetes and initiate timely treatment for optimal glycemic control, as evidence suggests that women seeking preconception medical care and achieving good glycemic control before pregnancy and in early pregnancy have fewer complications such as congenital malformations and perinatal mortality.^59^ Women of childbearing age visiting diabetes clinics, irrespective of pregnancy intention, should be given routine preconception counselling and information on the effective use of contraception and optimal timing of pregnancy.^60^ Dietary advice and weight reduction are also recommended for women with obesity.^61^, ^62^ Women also need to be informed of the risks and complications of GDM, and how these can be reduced. Postpartum follow‐up and glycemic evaluation of women with GDM are also of utmost importance.

## 8 Chronic Medical Conditions Before Pregnancy

Often women visit healthcare practitioners such as gynecologists and endocrinologists for issues related to subfertility and existing conditions such as thyroid disorders or PCOS. Screening, assessment, and management of such conditions should include evaluation of preconceptional endocrine issues. Women should be screened for hypertension before conception and those with hypertension should be informed about the risks of pre‐eclampsia and offered effective contraception if they so choose.^63^, ^64^ Women with PCOS should be screened for hypertension and diabetes and counselled for weight loss before conception (although there are limited data on the benefit of weight loss).^63^ Similarly, blood pressure should be optimally managed with medications adjusted to those appropriate for pregnancy prior to conception.

In many parts of the world, especially in low‐resource settings, repeated exposure to pollutants and toxic chemicals, such as endocrine disruptors, is common.^65^ These can accumulate in the maternal body to affect fetal growth and development. Some chemicals can also affect sperm quality. In these conditions, preconception counselling to limit exposure from potential airborne, food, and water sources is important, linked to wider political initiatives to reduce it.^66^

## 9 Incorporating Preconception Care Into Routine Maternal and Child Health Services

National preconception care guidelines often provide recommendations without focusing on women with unplanned pregnancies and identifying those at high risk of NCDs.^21^ While evaluation of screening for risk factors routinely is still needed, preconception care for planning pregnancy and achieving optimum nutrition has been recommended universally for all women and couples to prevent complications and NCD risks.^67^

Ideally, preconception care would comprise risk identification, education, and intervention provided by a range of healthcare practitioners, supplemented by specialist referrals where necessary.^40^ The four approaches for preconception healthcare delivery proposed by de Weerd^40^ include: universal primary care (opportunistic delivery within the primary care context via GPs, pharmacies, and nurses); hospital‐based opportunistic care (including interconception care after delivery); specialized preconception care clinics with targeted interventions; and high‐risk outreach preconception care.

Providing preconception care is the responsibility of all healthcare practitioners, especially in primary care, and is not limited to clinicians in maternity care. Family physicians and practitioners in primary care can also contribute to good quality preconception care, which is essentially good quality women’s health care and is an integral part of primary health care.^68^ Unplanned pregnancies can have adverse health and social consequences for mother and baby and addressing unmet contraceptive needs in routine primary care visits can help women at high risk. To help address reproductive needs of women in every clinical encounter, the Oregon Foundation for Reproductive Health^69^ developed the One Key Question strategy encouraging all healthcare practitioners to ask "Would you like to become pregnant in the next year?" to women in the reproductive age group. Based on the woman’s response (Yes/No/Unsure/OK either way) clinicians are encouraged to initiate a discussion on reproductive needs (preconception care or contraception) and provide appropriate preventive services.^70^ Preconception checklists, handouts, and tools for women explaining preconception care, available for example in the clinic waiting room, can improve preconception consultations.^27^ The FIGO Nutrition Checklist^49^, ^50^ can be modified for the local context and used to initiate a conversation related to nutrition before pregnancy and for risk assessment. Incorporating preconception care training in the educational curriculum for medical and allied health professions is also imperative.

Furthermore, efforts to tackle malnutrition must not be shouldered by healthcare practitioners alone but must be supported by public health policies for good nutrition, such as food fortification, and community engagement to increase physical activity and improve dietary habits.^13^ Public health policies such as food fortification with folic acid have led to around a 50% reduction in neural tube defects in many countries.^71^ Investment in national level community‐based interventions for preconception care show promise, such as China’s National Free Preconception Health Examination Project, which was expanded to all rural areas nationwide in 2013.^72^ The project included pregnancy planning and healthy lifestyle advice by trained staff and achieved more than 85% coverage of the population. Similarly, specific preconception care clinics have been established in several countries (e.g. Netherlands, Hungary) and their development requires prioritization by public health agencies and governments in other countries.^21^ Such preconception care programs for planning pregnancy and lifestyle modification before conception could be helpful in countries such as Colombia where there is increasing prevalence of adult and adolescent obesity.^73^ The Latin American region also has a high adolescent fertility rate (66.5 births per 1000 in those aged 15–19 years, compared to the global average of 46 births per 1000)^74^ and structured preconceptional education is crucial for such unplanned pregnancies.

## 10 Postnatal and Interconception Care

The postpartum period provides a platform for preparing for a possible next pregnancy and identifying women with, or at higher risk of, NCDs such as type 2 diabetes mellitus or GDM, addressing contraceptive needs, reversal of excess gestational weight gain, and addressing nutritional requirements.^21^, ^75^ For example, a study from the USA detected low rates of follow‐up glucose testing in women with GDM, especially in the postpartum period (5.8%), although this improved slightly after a year (21.8%) and at 3 years (51%).^76^ Contact with primary care after delivery was also very low. Several experts have called for recognizing the importance of, and providing, continuity of care in the “fourth trimester”^77^ taking into account the mother’s physical and mental health in the postpartum period.

Key service providers for infant health such as pediatricians, general practitioners, and health visitors have an important role in discussing interconception health, not only for the next pregnancy but also for the long‐term health of the mother. Not addressing these issues before the next pregnancy, especially for mothers with a history of outcomes such as low birthweight or medical comorbidities, is a missed opportunity for improving the woman’s health and outcomes of subsequent pregnancies.^75^ FIGO recommends the extension of preconception care into the postpartum stage to increase the window of opportunity and access women with nutritional needs, thus providing an integrated continuum of care for women.^1^, ^77^

Box 3 summarizes the key recommendations for preconception care in routine care that healthcare practitioners should consider, to support women in improving nutrition, weight management, and lifestyle before pregnancy.

Box 3 **Key messages for healthcare practitioners.**

1. Preconception care can and should be delivered in *any* clinical or hospital setting, regardless of the initial reason for a hospital visit, and at either an inpatient or outpatient level. Hospital‐based and community/home‐based opportunistic preconception care is recommended. This includes visits for contraception, immunization, and child health services.
2. A universal approach of discussing good nutritional habits, risks of obesity, and possible benefits of weight loss before pregnancy at every visit to a healthcare practitioner is recommended.
3. Failure to offer systematic advice and support regarding women's nutrition, weight, and related lifestyle behaviors in their childbearing years is a missed opportunity for preventing the intergenerational cycles of obesity and noncommunicable diseases.
4. As there is poor public understanding of the need for pregnancy planning and preparation in many countries, each contact with a healthcare practitioner is an opportunity to engage women in the reproductive age group in thinking about their health and understanding how their current diet, weight, and lifestyle will influence fertility, pregnancy outcomes, and the long‐term health and well‐being of their children.
5. Education and training of healthcare practitioners must be addressed urgently, to ensure development of skills and confidence in raising nutrition and weight management issues with women, including training for motivational interviewing and “healthy conversation” skills. Healthcare organizations should give attention to the dissemination of clinical guidelines for preconception weight and nutrition management, and developing, using, and adapting simple tools for clinical practice.
6. Interconception care: healthcare practitioners such as general practitioners, pediatricians, and those involved in postnatal care such as home visitors have an important role to play in interconception care (e.g. a follow‐up test for HBA1C, weight management, physical exercise, and nutrition advice).
7. Healthcare practitioners should be cognizant of the common nutritional deficiencies of different age groups. When approached by women in the preconception period they should discuss any dietary restrictions and screen and treat nutritional deficiencies following local dietary guidelines. Where applicable, healthcare practitioners in a variety of settings are encouraged to adapt and use the FIGO Nutrition Checklist to initiate conversations on nutrition and weight management during appointments, and to identify women requiring further intervention or referral to a nutritionist or dietitian.

## 11 Conclusion

The FIGO Pregnancy and Non‐Communicable Diseases Committee and the FIGO Committee for Reproductive Medicine, Endocrinology, and Infertility recommend that all healthcare practitioners engage with the provision of systematic advice and support for women’s nutrition and weight management in the preconception period in a supportive and nonstigmatizing manner. Addressing the barriers to communication and using tools such as the FIGO Nutrition Checklist to hold effective discussions in short clinical visits can have long‐lasting effects for women’s health, pregnancy outcomes, and the long‐term health of the offspring. Healthcare practitioners should prioritize attention to common comorbidities and nutritional issues in their settings, underpinned by the FIGO preconception recommendations in this paper, to ensure all women think about nutrition, weight management, and lifestyle after each consultation and feel empowered to make any necessary changes. Other stakeholders such as public health organizations and professional organizations have a key role to play in addressing training needs and ensuring that the recommendations of these guidelines become embedded throughout their organizations.

## Author Contributions

This position paper was developed by the FIGO Pregnancy and Non‐Communicable Diseases Committee in collaboration with the FIGO Committee for Reproductive Medicine, Endocrinology, and Infertility. All authors were involved in the conception and design of the paper. CMJ wrote the manuscript and all authors provided input into the revisions of the manuscript.

## Conflicts of Interest

The authors have no conflicts of interest.

## Supporting information


**Supporting information S1**. Guidelines relevant to nutrition and prevention of obesity and noncommunicable diseases in the preconception period reviewed by the FIGO Pregnancy and Non‐Communicable Diseases Committee and the FIGO Committee for Reproductive Medicine, Endocrinology, and Infertility.

**Supporting information S2**. FIGO nutrition checklist for pre‐pregnant/early pregnant women. Reproduced with permission from FIGO.
