# Supplementary material for: Prevention of noncommunicable diseases by interventions in the preconception period: A FIGO position paper for action by healthcare practitioners
Source: Int J Gynaecol Obstet. 2020 Sep 7;151(Suppl 1):6–15. doi: 10.1002/ijgo.13331 (PMC7590173; doi:10.1002/ijgo.13331)
Supplement: Supplementary file 1 — Supporting information S1. Guidelines relevant to nutrition and prevention of obesity and noncommunicable diseases in the preconception period reviewed by the FIGO Pregnancy and Non‐Communicable Diseases Committee and the FIGO Committee for Reproductive Medicine, Endocrinology, and Infertility. [file IJGO-151-6-s001.docx]

**Supporting information S1:** Guidelines relevant to nutrition and prevention of obesity and noncommunicable diseases in the preconception period reviewed by the FIGO Pregnancy and Non-Communicable Diseases Committee and the FIGO Committee for Reproductive Medicine, Endocrinology, and Infertility. Recommendations were extracted from documents published by key maternal and reproductive health organizations and relevant systematic reviews. To ensure the latest guidelines were adopted, only documents published in the last 10 years were considered.

| **Citation/organization, Year** | **Target population** | **Key clinical recommendations for the preconception period ^a^** |
| --- | --- | --- |
| **Australia** |  |  |
| Australia: Royal Australian College of General Practitioners (RACGP) [1] | General preconception care | Regional organizations such as South Australia’s Preconception Advice Clinical Guideline have developed comprehensive online resources to assist the pre-pregnancy counselling process.  The preconception care checklist includes:  **Diet**  Nutritional requirements including folic acid supplementation  Advice on a healthy diet  **Weight**  Measurement of body mass index and appropriate advice  **Exercise**  Advise 150 minutes of exercise per week or 30 minutes on most days  **Pregnancy history**  Screen for any modifiable risk factors |
| The Royal Australian and New Zealand College of Obstetricians and Gynaecologists (RANZCOG) (2017) [2] | Target audience: All health professionals providing care to women prior to pregnancy. | The document provides health professionals with advice on the counselling of women prior to pregnancy.  All women planning a pregnancy are advised to consult their General Practitioner with a view to: (1) detecting and assessing any specific health problems in the woman or her partner that may be relevant, so that these can be appropriately managed prior to the pregnancy; (2) obtaining general advice about optimizing personal health care and lifestyle with pregnancy in mind. Other healthcare professionals (such as obstetricians, infertility specialists, and midwives), may also be presented with a valuable opportunity to assess and counsel a woman prior to a planned pregnancy.  **Lifestyle recommendations**:   - Healthy weight - Active steps to correct high BMI (dietary, exercise and where appropriate consideration of bariatric surgery) prior to a pregnancy should be recommended. - A recommendation for moderate intensity exercise and assessment of any nutritional deficiencies is appropriate. - Excessive caffeine consumption (>300 mg/day; equivalent to 3–4 cups of brewed coffee/day) should be avoided - Supplementation - Folic acid should be taken for a minimum of one month before conception and for the first 3 months of pregnancy. The recommended dose is at least 0.4 mg daily. Where there is an increased risk of neural tube defects (anticonvulsant medication, prepregnancy diabetes mellitus, previous child or family history of neural tube defects, BMI >35), a 5 mg daily dose should be used.   The National Health and Medical Research Council recommends women should start a dietary supplementation of 150 µg iodine prior to a planned pregnancy or as soon as possible after finding out they are pregnant. |
| **Canada** |  |  |
| College of Family Physicians of Canada [3] |  | The Public Health Agency of Canada published preconception guidelines in 2000, urging that “preconception care and education be incorporated into school curricula and the workplace, delivered through the media, and offered through community-based agencies.” Though national guidelines are still lacking, steps have been taken at the level of provinces. |
| Public Health Agency of Canada (2018) [4] | Information for HCPs to optimize preconception health | The goals for preconception care are to improve the health status of women and men before conception and to reduce those behaviors and individual and environmental factors that could contribute to poor maternal and child health outcomes.  Key recommendations include:   - Encourage all women and men of reproductive age to develop a reproductive-life plan, whether they intend to have children or not. - Recommend a daily multivitamin containing 400µg (0.4 mg) of folic acid for all women of reproductive age who could become pregnant and discuss risk factors that may warrant a higher dose. - Encourage progress toward healthier weights in women who are underweight, overweight, or obese. Adverse perinatal and maternal outcomes can be reduced with appropriate preconception weight gain or loss. - Optimize chronic medical conditions prior to conception to improve perinatal and maternal outcomes. - Obese women should be made aware that a weight loss of as little as 5%–10% of their current weight can improve their chances of conceiving - Lifestyle advice: - 150 minutes per week of moderate to vigorous physical activity for adults aged 64 and under. - No more than 2 drinks per day on most days, with no more than 10 drinks per week for nonpregnant women to reduce long-term health risks. - All women who could become pregnant should take a daily multivitamin containing 400 µg (0.4 mg) of folic acid - A pre-existing pattern of healthy eating helps to optimize maternal and fetal health. Advice for avoiding fad diets and the need to counsel women about having a healthy balanced diet is included in the report.   The preconception period is the ideal time to achieve (or progress toward) an optimal weight |
| **China** |  |  |
| China’s National Preconception Health Care Project (NPHCP)  Zhou et al. 2016 [5] |  | The national program mainly targeted communities with a focus on healthy weight and nutrition and improving diet. However, as obesity rates vary across regions more action has been called for preventing weight gain before and during pregnancy, to increase awareness among underweight and overweight women. |
| **Ireland** |  |  |
| Institute of Obstetricians and Gynaecologists, Royal College of Physicians of Ireland 2013 [6] | Obesity and pregnancy clinical practice guideline | - It is recommended that obese women should take high dose (5 mg) folic acid for at least one month before conception and continue throughout the first trimester. - Women of childbearing age who are overweight or obese should be encouraged to lose weight whether they plan to conceive or not.   Obesity is associated with polycystic ovarian syndrome and women with obesity and anovulation who are planning a pregnancy are more likely to conceive if they lose weight although there is little evidence that one diet is better than another for enhancing reproduction. |
| **India** |  |  |
| Federation of Obstetric and Gynaecological Societies of India (FOGSI)  Good Clinical Practice Recommendations on Preconception Care (2016) [7] | For healthcare practitioners | Comprehensive set of recommendations for clinical practice for women in the preconception period:  **Folic acid:**  All women of childbearing age should be recommended to take folic acid 0.4/0.5 mg daily, at least 1 month before conception to up to 3 months after conception to reduce the risk of neural tube defects.  **Anemia**: All women in preconception period should be screened for anemia with hemoglobin as a primary screening test and treated appropriately. In addition, considering a very high incidence of anemia in India, weekly supplementation of 100 mg elemental iron and 500 µg folic acid with deworming medication (albendazole 400 mg) should be recommended to all women in the preconception period.  **BMI:** **It is advisable to attain BMI 18–23 prior to conception and healthcare providers should advise women about the measures to attain it.**  BMI cut-off for Asian Indians: Normal BMI: 18.0–22.9; Overweight: 23.0–24.9; Obesity: >25.  **Overweight:** Overweight and obese women in the preconception period should be counselled about the increased risk of adverse maternal and perinatal outcomes.  Focused counselling sessions combined with multipronged interventions consisting of nutritional modification along with aerobic and strength-conditioning exercises should be the first-line approach to achieve the target weight loss.  • Irrespective of the pre-pregnancy weight, weight loss during pregnancy is not recommended and hence counselling during preconception should be done to achieve a realistic target of 5%–10% over a period of 6 months.  • Bariatric surgery is suggested in women with BMI above 32.5 with comorbidities, and in women with BMI above 37.5 without comorbidities. Patients should be advised to avoid pregnancy for at least 12–18 months after the surgery.  **Underweight:** Healthcare providers should examine the food choices and provide nutritional advice to underweight women.  • Underweight women should also be screened and treated for eating disorders like anorexia nervosa and bulimia.  • Counselling about proper nutrition to maintain optimal BMI well before pregnancy should be provided as weight gain in pregnancy does not reduce the risks associated with the low pre-pregnancy BMI.  **Diabetes:**   - In the preconception period, all women should be screened for diabetes as per WHO criteria   **(**Fasting plasma glucose (FPG) ≥126 mg/dL or 2-hr plasma glucose ≥200 mg/dL)  • All women with pregestational diabetes should be counselled on diabetes self-management skills, the importance of maintaining good glycemic control before and throughout pregnancy, and about the strong benefits of long-term cardiovascular disease risk factor reduction.  • Women with pre-existing diabetes mellitus should be advised to achieve a glucose level of 80–110 mg/dL (fasting) and an HbA1c goal of <6.5% before conception. |
| **United Kingdom** |  |  |
| NICE 2015  United Kingdom [8] | Clinicians in contact with women of childbearing age with diabetes and for gestational diabetes risk assessment | - Offer women with diabetes who are planning to become pregnant individualized dietary advice. - Offer women with diabetes who are planning to become pregnant and who have a BMI above 27 advice on how to lose weight, in line with the NICE guideline on obesity: identification, assessment, and management of overweight and obesity in children, young people, and adults. - Advise women with diabetes who are planning to become pregnant to take folic acid (5 mg/day) until 12 weeks of gestation to reduce the risk of having a baby with a neural tube defect. - Contraception and planning for pregnancy. - Providing information, advice, and support on outcomes for mother and baby. - Offer women with diabetes who are planning to become pregnant monthly measurement of their HbA1C level. - Offer women with diabetes who are planning to become pregnant a meter for self-monitoring of blood glucose. - Review safety of current medication. - Retinal and renal assessment.   Also includes: Removing barriers to the uptake of preconception care and when to offer information.  1.1.26 Explain to women with diabetes about the benefits of preconception blood glucose control at each contact with healthcare professionals, including their diabetes care team, from adolescence.  1.1.27 Document the intentions of women with diabetes regarding pregnancy and contraceptive use at each contact with their diabetes care team from adolescence.  1.1.28 Ensure that preconception care for women with diabetes is given in a supportive environment and encourage the woman's partner or other family member to attend. |
| NICE 2019  United Kingdom [9] | Preconception advice and management | Overall advice on diet:   - **Using the Eatwell guide for information on achieving a balance of healthier food** - **Dietary advice to women planning pregnancy to eat a healthy, balanced diet and to help maintain a healthy weight before pregnancy (recommendations include, for example - b**ase meals on starchy food (for example bread, rice, pasta, potatoes), choosing wholegrain if possible, eat fiber-rich foods (for example fruit, vegetables, oats, beans, peas, lentils), eat at least 5 portions of different fruits and vegetables each day, low fat diet, reduced consumption of fried food, drinks and confectionary with added sugar (for example cakes, fizzy drinks) etc. - Advise women that achieving a healthy weight (BMI 18.5–24.9) before becoming pregnant reduces the risk of pregnancy complications. - Advise the woman of the potential health risks of being obese (BMI of 30 or more). - **Advise on achieving a healthy weight before conception and risks of being overweight before conception** - Weight loss - the recommendation to advise women to lose weight if obese, and the target weight loss suggested is based on the NICE public health guidance: *Weight management before, during and after pregnancy.* - **Folic acid:** recommendation to prescribe folic acid 5 mg daily to people at higher risk of neural tube defects and 400 µg to people at normal risk of neural tube defects. - **Counsel about risk of being underweight before conception.** - **Managing suspected eating disorders** |
| RCOG 2018, UK  Green-top Guideline No. 72 [10] | Women with obesity in pregnancy | - Primary care services should ensure that all women of childbearing age have the opportunity to optimize their weight before pregnancy. Advice on weight and lifestyle should be given during preconception counselling or contraceptive consultations. Weight and BMI should be measured to encourage women to optimize their weight before pregnancy. - Women of childbearing age with a BMI 30 or greater should receive information and advice about the risks of obesity during pregnancy and childbirth and be supported to lose weight before conception and between pregnancies in line with National Institute for Health and Care Excellence (NICE) Clinical guideline (CG) 189. - Women should be informed that weight loss between pregnancies reduces the risk of stillbirth, hypertensive complications, and fetal macrosomia. Weight loss increases the chances of successful vaginal birth after cesarean section. - **Nutritional supplements**: Women with a BMI 30 or greater wishing to become pregnant should be advised to take 5 mg folic acid supplementation daily, starting at least 1 month before conception and continuing during the first trimester of pregnancy.   Women with obesity are at high risk of vitamin D deficiency. However, although vitamin D supplementation may ensure that women are vitamin D replete, the evidence on whether routine vitamin D should be given to improve maternal and offspring outcomes remains uncertain. |
| RCOG – Better for Women report 2019 [11] |  | - Overall, the report highlights the importance of a life-course approach. - Health services need to find innovative ways of communicating with girls and women at an early stage in their reproductive lives, to highlight the importance of eating a healthy diet, having a normal BMI, being physically active, stopping smoking, avoiding alcohol and recreational drugs, and taking supplements of folic acid in the preconception period. - Data collection should be supported before, during, and after pregnancy and maternity and SRH data can be integrated by the inclusion of outcomes such as the London Measure of Unplanned Pregnancy in the antenatal booking history, which can be used to monitor the effectiveness of preconception and family planning services and identify areas for action. - Each contact with healthcare practitioner is an educational opportunity to engage women in thinking about their health, preparing for pregnancy, and understanding how their current lifestyle choices will influence the outcome of their pregnancy and their baby’s future health – these include attending their GP practice or gynecology clinic for the insertion or removal of a contraceptive implant or intrauterine device, or visiting their GP or local early pregnancy unit after a miscarriage or ectopic pregnancy, baby checks, and vaccination visits for young children.   Women should be offered advice on body weight and lifestyle in primary care, such as during preconception counselling and appointments about contraception. |
| **USA** |  |  |
| American Academy of Family Physicians (AAFP) 2016  Preconception Care [12] | General practitioners | - All women who have a BMI greater than 30 or less than 18.5 should be counselled about the risks their weight status poses to their own health and to future pregnancies; these patients should be offered specific strategies to improve the balance and quality of their diet and physical activity level. |
| ACOG 2019  Prepregnancy counselling [13] | OBGYNs and other healthcare practitioners | - Prepregnancy counselling is recommended whether the reproductive-aged patient is currently using contraception or planning pregnancy. Counselling can begin with the following question: “*Would you like to become pregnant in the next year*?” - Any patient encounter with nonpregnant women or men with reproductive potential is an opportunity to counsel about wellness and healthy habits, which may improve reproductive and obstetric outcomes should they choose to reproduce. - Patients should be screened regarding their diet and vitamin supplements to confirm they are meeting recommended daily allowances for calcium, iron, vitamin A, vitamin B_12_, vitamin B, vitamin D, and other nutrients. - Patients should be encouraged to try to attain a BMI in the normal range before attempting pregnancy, because abnormal high or low BMI is associated with infertility and maternal and fetal pregnancy complications. Ideally, weight should be optimized before a woman attempts to becoming pregnant, although the health benefits of postponing pregnancy need to be balanced against reduced fecundity with female aging. - Prepregnancy, during pregnancy, and postpartum - women should exercise moderately at least 30 minutes a day, 5 days a week, for a minimum of 150 minutes of moderate exercise per week. These levels of exercise are recommended. - Pregestational diabetes: Euglycemic control (HbA1C <6.5%) and optimal weight management. Additionally, screen for thyroid dysfunction |
| American Association of Clinical Endocrinologists [14] | Women with PCOS | - Assessment of a woman with PCOS for infertility involves evaluating for preconception issues that may affect response to therapy or lead to adverse pregnancy outcomes and evaluating the couple for other common infertility issues that may affect the choice of therapy, such as a semen analysis. Women with PCOS have multiple factors that may lead to an elevated risk of pregnancy, including a high prevalence of IGT (a clear risk factor for gestational diabetes) and MetS with hypertension, which increases the risk for pre-eclampsia and placental abruption. Women should be screened and treated for hypertension and diabetes prior to attempting conception. Women should be counseled about weight loss prior to attempting conception, although there are limited clinical trial data demonstrating a benefit to this recommendation. Treatment for women with PCOS and anovulatory infertility should begin with an oral agent such as clomiphene citrate or letrozole, an aromatase inhibitor. |
| **Guidelines by the International Health Organizations** | | |
| World Health Organization 2013 [15] |  | - Folic acid insufficiency, anemia, and iron deficiency: Iron and folic acid supplementation (e.g. food fortification, use of micronutrient powders containing iron); screening for anemia; information; education. - Underweight: Nutrition education (counselling about risks to own health and future pregnancies); nutritional monitoring. - Overweight and obesity: Nutrition education (counselling about risks to own health and future pregnancies); nutritional monitoring; nutrition counselling (lower caloric intake, increase physical activity, structured weight-loss program, continued breastfeeding). - Diabetes mellitus: Information and education; communitywide or national screening among populations at high risk; blood glucose monitoring; management of diabetes (glycemic control before, during, and after pregnancy); exercise; nutritional counselling (screening for pre-existing type 2 diabetes and every 1–3 years after gestational diabetes).   Iodine deficiency: however clinical intervention is not recommended (iodized salt as a public health measure recommended). |
| WHO Regional Office for South-East Asia (2014) [16] | WHO South East Asia region | - Folic acid deficiency, anemia and underweight were identified as important nutritional issues for preconception care along with increasing maternal overweight and obesity.   Specific clinical recommendations not mentioned. |
| FIGO (International Federation of Gynecology and Obstetrics)  Hanson et al. (2015) “Think Nutrition first” [17] and FIGO Working Group on Good Clinical Practice in Maternal–Fetal Medicine (2019) Good clinical practice advice: Micronutrients in the periconceptional period and pregnancy [18] | Women in the reproduction age group | - Women who plan to become pregnant or who are of childbearing age and not using a contraceptive method, and women who do not have risk factors for neural tube defects, should take 400 µg (0.4 mg) of synthetic folic acid beginning at least 30 days before conception and continue daily supplements throughout the first trimester of pregnancy. - High risk pregnancies for neural tube defects:   Those with a personal or family history of pregnancy affected by some neural tube defects, use of anticonvulsants, mutation in enzymes related to folate (e.g. *MTHFR*), insulin dependent diabetes, obesity (body mass index >30), malabsorption syndromes, and pregnant women with a history of surgery for obesity. Such high-risk groups should be advised to take 4000 µg per day (4.0 mg). For both regimens, supplementation must begin at least 30 days before conception and be continued daily throughout the first trimester of pregnancy.   - Patients should be asked about their use of iodized salt and be informed of the importance of adequate iodine nutrition to ensure optimal thyroid function both before and during pregnancy. - Attention should be paid to preconception or early pregnancy body weight and BMI, and steps should be taken to provide adequate support for dietary modification and achieving appropriate levels of physical activity. - General preconception and adolescent health assessment by healthcare providers can include as appropriate for the setting: diet composition, physical activity, height, weight, BMI, obesity risk – waist circumference and other anthropometric measures, anemia, risk of specific nutritional problems (low nutrient density) folate, iron, calcium, vitamin B12, vitamin D, iodine, zinc, PUFAs.   For women specifically planning a pregnancy, this could include discussions related to: Importance of a healthy diet and exercise, sedentary behavior, weight loss counselling, risky behaviors and exposures (e.g. tobacco, alcohol, recreational drugs), environmental toxins, chronic disease screening and management, supplementation. Folic acid supplementation 400 µg/day and other nutrients as required (iron, iodine, vitamin B12). |
| **Other key publications on preconception weight management and NCD prevention** | | |
| Shannon et al*.* (2014) [19] | Public health models of preconception healthcare delivery | Based on the systematic review, four approaches were adopted in developing a model of healthcare delivery:   1. Universal, primary care, defined as the provision of preconception healthcare opportunistically within the context of primary healthcare services such as general practice, nurse practitioners, local healthcare clinics, and pharmacies. 2. Hospital-based opportunistic care, including education, interconception care. Using a hospital admission as a point of contact with the healthcare system and a platform for general patient medical education. 3. Establishment of specialized preconception healthcare clinics, which provide targeted, specific preconception healthcare. 4. High-risk and outreach preconception care, defined as the identification of women with specific medical and social issues that may be of higher risk during pregnancy than the general population, and targeting this group with appropriate interventions, including community outreach. |
| Shawe et al*.* (2015) [20] | Review of Guidelines in Six European Countries | - Overall, preconception care recommendations were available for women with chronic disease but guidance for healthy women was fragmented and inconsistent, and there was very little guidance relating to men. In all countries, antenatal care and pregnancy guidelines were found that alluded to the requirement for good health before pregnancy and included advice about folic acid supplementation before conception. - At the time of review, only Italy, the Netherlands, and the UK had definitive preconception national guidelines available via the web to healthcare practitioners for women without pre-existing medical conditions. - UK, Netherlands, Italy, and Sweden had specific dietary and nutritional advice. - In the UK and Belgium, guidelines for women planning pregnancy included counselling about the associated risks and recommend structured weight loss programs for women with a BMI exceeding 30 and that healthcare practitioners should actively discuss this issue with women of childbearing age. |
| Simon et al. (2020) [21] | Systematic review of clinical practice guidelines for the management of pregnant women with obesity | **Recommendations for preconception care of women in childbearing age with obesity**   - Women of childbearing age with obesity should receive information from healthcare providers about both the risks of being affected by obesity and the benefits of weight loss before pregnancy, specifically improving pregnancy outcomes for both mother and baby by reducing the risks of miscarriage, pre-eclampsia, and gestational diabetes mellitus (GDM). - Women should be reminded that weight loss also reduces long-term health risks, including hypertension, sleep apnea, pulmonary disease, and cardiac disease especially in women with diabetes. - Women with a BMI ≥30 wishing to become pregnant should be advised to take 5 mg of folic acid supplementation daily, starting at least 1 month before conception and continuing during the first trimester of pregnancy. - In addition, prior to attempting to conceive, women of childbearing age should stop taking medication for weight loss. - One clinical practice guideline (CPG) recommended that “bariatric surgery could be considered to improve fertility outcomes in women with PCOS who are anovulatory, have a BMI ≥35 kg/m2, and who remain infertile despite undertaking an intensive structured lifestyle management programme involving reducing dietary energy intake, exercise, and behavioural interventions preferably for a minimum of 6 months.” However, other CPGs have suggested that bariatric surgery should not be considered as a treatment for infertility. One CPG recommended that if a woman has had bariatric surgery prepregnancy, ongoing follow-up by a dietician is necessary. |
| Zühlke et al. (2016) [22] | Recommendations for preconception counselling in the African context | - Based on WHO recommendations for preconception counselling (2013) around 13 areas and provide an evidence-based package of interventions addressing the following areas: nutritional conditions; vaccine-preventable diseases; genetic conditions; environmental health; infertility/subfertility; female genital mutilation; too early, unwanted, and rapid successive pregnancies; sexually transmitted infections; HIV; interpersonal violence; mental health; psychoactive substance abuse; and tobacco use. - Evidence-based interventions to be considered for: screening for anemia, nutritional supplementation (iron and folate), information, education and counselling, food supplementation, promoting exercise and a healthy diet, and family planning and child spacing. - Components of preconception care should include nutritional assessment. - When possible, prepregnancy blood pressure should be normalized with lifestyle changes before pregnancy such as dietary changes (low salt intake, increased intake of fresh fruits and vegetables), healthy weight modification to avoid obesity, and adherence to antihypertensive medications. |

^a^ Information with focus on nutrition and noncommunicable disease prevention (e.g. weight loss, diet, and physical activity) is listed here. For further details please refer to the original reference.

**References**

1. Dorney E, Black KI. Preconception care. Aust J Gen Pract.2018;47(7):424–429.

2. Royal Australian and New Zealand College of Obstetricians and Gynaecologists. Pre-pregnancy Counselling. July 2017. <https://ranzcog.edu.au/RANZCOG_SITE/media/RANZCOG-MEDIA/Women%27s%20Health/Statement%20and%20guidelines/Clinical-Obstetrics/Pre-pregnancy-Counselling-(C-Obs-3a)-review-July-2017_1.pdf?ext=.pdf>. Accessed May 15. 2020.

3. Bialystok L, Poole N, Greaves L. Preconception care: call for national guidelines [in French]. Can Fam Physician. 2013;59(10):1037–9.

4. Public Health Agency of Canada. Chapter 2: Preconception Health. 2019. <https://www.canada.ca/content/dam/phac-aspc/documents/services/publications/healthy-living/maternity-newborn-care/maternity-newborn-care-guidelines-chapter-2-eng.pdf>. Accessed May 15, 2020.

5. Zhou Q, Zhang S, Wang Q, Shen H, Tian W, Chen J, et al. China’s community-based strategy of universal preconception care in rural areas at a population level using a novel risk classification system for stratifying couples’ preconception health status. BMC Health Serv Res 2016;16(1):689.

6. Institute of Obstetricians and Gynaecologists, Royal College of Physicians of Ireland and Clinical Strategy and Programmes Directorate, Health Service Executive. Obesity and Pregnancy Clinical Practice guideline Revised June 2013. <https://www.hse.ie/eng/services/publications/clinical-strategy-and-programmes/obesity-and-pregnancy-clinical-practice-guideline.pdf>. Accessed May 15, 2020.

7. The Federation of Obstetric and Gynaecological Societies of India. Good Clinical Practice Recommendations on Preconception Care 2016. https://www.fogsi.org/gcpr-preconception-care/. Accessed May 15, 2020.

8. National Institute for Health and Care Excellence. Diabetes in pregnancy: management from preconception to the postnatal period. NICE guideline [NG3]. Published February 25, 2015. https://www.nice.org.uk/guidance/ng3. Accessed May 15, 2020.

9. National Institute for Health and Care Excellence. Pre-conception - advice and management. November 2019. https://cks.nice.org.uk/pre-conception-advice-and-management. Accessed May 15, 2020.

10. Royal College of Obstetricians and Gynaecologists. Care of Women with Obesity in Pregnancy (Green-top Guideline No. 72) Published November 22, 2018. https://www.rcog.org.uk/en/guidelines-research-services/guidelines/gtg72/. Accessed May 15, 2020.

11. Royal College of Obstetricians and Gynaecologists. Better for Women: Improving the health and wellbeing of girls and women. December 2019. https://www.rcog.org.uk/globalassets/documents/news/campaigns-and-opinions/better-for-women/better-for-women-full-report.pdf. Accessed May 15, 2020.

12. American Academy of Family Physicians. Preconception care (Position Paper) 2016. <https://www.aafp.org/about/policies/all/preconception-care.html>. Accessed May 15, 2020.

13. ACOG Committee Opinion No. 762; Prepregnancy Counseling. Obstet Gynecol. 2019;133:e78–e89.

14. Goodman NF, Cobin RH, Futterweit W, et al. American Association of Clinical Endocrinologists, American College of Endocrinology, and Androgen Excess and PCOS Society disease state clinical review: guide to the best practices in the evaluation and treatment of polycystic ovary syndrome-part 2. Endocr Pract. 2015;21(12):1415–26.

15. World Health Organization. Preconception care to reduce maternal and childhood mortality and morbidity. Geneva: WHO; 2013. <https://www.who.int/maternal_child_adolescent/documents/concensus_preconception_care/en/>. Accessed May 15, 2020.

16. World Health Organization Regional Office for South-East Asia. Preconception care. 2014. <https://apps.who.int/iris/handle/10665/205637>. Accessed May 15 202017. Hanson MA, Bardsley A, De-Regil LM, et al. The International Federation of Gynecology and Obstetrics (FIGO) recommendations on adolescent, preconception, and maternal nutrition:"Think Nutrition First". Int J Gynecol Obstet. 2015;131(Suppl 4):S213–53.

18. FIGO Working Group on Good Clinical Practice in Maternal–Fetal Medicine. Good clinical practice advice: Micronutrients in the periconceptional period and pregnancy. Int J Gynecol Obstet. 2019;144(3):317–21.

19. Shannon GD, Alberg C, Nacul L, Pashayan N. Preconception healthcare delivery at a population level: construction of public health models of preconception care. Matern Child Health J. 2014;18(6):1512–31.

20. Shawe J, Delbaere I, Ekstrand M, et al. Preconception care policy, guidelines, recommendations and services across six European countries: Belgium (Flanders), Denmark, Italy, the Netherlands, Sweden and the United Kingdom. Eur J Contracept Reprod Health Care 2015;20(2):77–87.

21. Simon A, Pratt M, Hutton B, et al. Guidelines for the management of pregnant women with obesity: A systematic review. Obes Rev. 2020;21(3):e12972.

22. Zühlke L, Acquah L. Pre-conception counselling for key cardiovascular conditions in Africa: optimising pregnancy outcomes. Cardiovasc J Afr. 2016;27(2):79–83.
